# The 121 amino acid isoform of vascular endothelial growth factor is more strongly tumorigenic than other splice variants in vivo

**DOI:** 10.1054/bjoc.2000.1279

**Published:** 2000-06-02

**Authors:** H-T Zhang, P A E Scott, L Morbidelli, S Peak, J Moore, H Turley, A L Harris, M Ziche, R Bicknell

**Affiliations:** Molecular Angiogenesis Laboratory, Imperial Cancer Research Fund, Institute of Molecular Medicine, University of Oxford, John Radcliffe Hospital, Oxford, UK; Department of Oncology, Christchurch Hospital, Christchurch, New Zealand; Department of Pharmacology, University of Florence, Florence, Italy; Clare Hall Laboratories, Imperial Cancer Research Fund, South Mimms, Herts, UK; Department of Cellular Science, University of Oxford, John Radcliffe Hospital, Oxford, UK; Institute of Pharmacological Science, School of Pharmacy, University of Siena, Via Piccolomini 170, Siena, 53100, Italy

**Keywords:** vascular endothelial growth factor, angiogenesis, tumorigenesis, differential splicing, nuclear localization

## Abstract

Vascular endothelial growth factor (VEGF) is known to occur as at least six differentially spliced variants, giving rise to mature isoforms containing 121, 145, 165, 183, 189 and 206 amino acids. However, little is yet known concerning the in vivo activities of this differential splicing. Stably transfected MCF-7 breast carcinoma cells were constructed that secreted comparable amounts of the 121, 165 or 189 isoforms. Rabbit corneal angiogenesis assays showed the VEGF121 transfectant to have much greater angiogenic activity than the 165 or 189 expressing MCF-7 cells. While the VEGF121-expressing MCF-7 cells were reproducibly more tumorigenic than the control transfectants, this was not the case with the VEGF165- or VEGF189-expressing cells. More surprising was the observation that VEGF189 located to the nucleus, consistent with the presence of a highly conserved nuclear localization sequence in exon 6a that is expressed in VEGF189 but not 121 or 165. It was concluded that the VEGF121 isoform is both more angiogenic and tumorigenic than are the 165 and 189 isoforms. This is probably due to the ability of the 121 isoform, unlike the 165 and 189 isoforms, to freely diffuse from the cells producing it. © 2000 Cancer Research Campaign


*British Journal of Cancer* (2000) **83**(1), 63–68
© 2000 Cancer Research Campaign
doi: 10.1054/ bjoc.2000.1279, available online at http://www.idealibrary.com on IDEAL®

### The 121 amino acid isoform of vascular endothelial growth factor is more strongly tumorigenic than other splice variants in vivo

**H-T Zhang[1], PAE Scott[2], L Morbidelli[3], S Peak[4], J Moore[1], H Turley[5], AL Harris[1], M Ziche[6] and R Bicknell[1]**

[1]Molecular Angiogenesis Laboratory, Imperial Cancer Research Fund, Institute of Molecular Medicine, University of Oxford, John Radcliffe Hospital, Oxford, UK; [2]Department of Oncology, Christchurch Hospital, Christchurch, New Zealand; [3]Department of Pharmacology, University of Florence, Florence, Italy; [4]Clare Hall Laboratories, Imperial Cancer Research Fund, South Mimms, Herts, UK; [5]Department of Cellular Science, University of Oxford, John Radcliffe Hospital, Oxford, UK; [6]Institute of Pharmacological Science, School of Pharmacy, University of Siena, Via Piccolomini 170, 53100 Siena, Italy

**Summary** Vascular endothelial growth factor (VEGF) is known to occur as at least six differentially spliced variants, giving rise to mature isoforms containing 121, 145, 165, 183, 189 and 206 amino acids. However, little is yet known concerning the in vivo activities of this differential splicing. Stably transfected MCF-7 breast carcinoma cells were constructed that secreted comparable amounts of the 121, 165 or 189 isoforms. Rabbit corneal angiogenesis assays showed the VEGF121 transfectant to have much greater angiogenic activity than the 165 or 189 expressing MCF-7 cells. While the VEGF121-expressing MCF-7 cells were reproducibly more tumorigenic than the control transfectants, this was not the case with the VEGF165- or VEGF189-expressing cells. More surprising was the observation that VEGF189 located to the nucleus, consistent with the presence of a highly conserved nuclear localization sequence in exon 6a that is expressed in VEGF189 but not 121 or 165. It was concluded that the VEGF121 isoform is both more angiogenic and tumorigenic than are the 165 and 189 isoforms. This is probably due to the ability of the 121 isoform, unlike the 165 and 189 isoforms, to freely diffuse from the cells producing it.
© 2000 Cancer Research Campaign

**Keywords:** vascular endothelial growth factor; angiogenesis; tumorigenesis; differential splicing; nuclear localization

Vascular endothelial cell growth factor (VEGF) is now known to be a key factor in the induction of angiogenesis in many tumour types (reviewed in Neufeld et al, 1999). VEGF has for several years been known to exist as several isoforms arising from differential splicing of the mRNA. Human VEGF mRNA is transcribed from 8 exons and alternatively spliced into at least five variants which give rise to mature monomers of 121, 145, 165, 189 and 206 amino acids (Tischer et al, 1991). Recently, a new splice variant of 183 amino acids has been identified (Lei et al, 1998). While it has also been known for several years that the isoforms show little difference in their affinity for the VEGF receptors, flt-1 and KDR, it is known that they differ in the affinity with which they bind to heparin and heparin-like molecules. Thus, the larger isoforms (165 and 189) bind tightly to heparin, while the smaller 121 isoform does not (Houck et al, 1992). It has been reported that the 121 amino acid isoform is a 100-fold less potent mitogen than the 165 or 189 isoforms (Keyt et al, 1996), but this has been questioned by others (Gitay-Goren et al, 1996; Siemeister et al, 1996).

Of the isoforms, expression of VEGF206 is rare, occurring predominantly in fetal liver (Houck et al, 1991). In contrast, the mRNAs corresponding to the 121, 165, and 189 isoforms are found in most normal tissues, VEGF121 and VEGF165 predominate in normal tissue (Houck et al, 1991). Increasingly, however, exceptions to this uniform pattern of variant mRNA expression are emerging. These include differential patterns of expression in tissues (Carmeliet et al, 1999) as well as activation-dependent cell-specific expression of VEGF isoforms. In this regard, VEGF189 expression has been shown to be dominant in normal lung (Cheung et al, 1998) and the 183 isoform predominates in heart (Lei et al, 1998). VEGF206 has been detected in skin and HMC-1 mast cells after a prolonged stimulation with phorbol-12-myristate-13-acetate and the ionophore A23187 (Grutzkau et al, 1998). Selective knockout of the 165 and 189 isoforms using cre/lox recombinant technology in mice has shown that the higher molecular weight isoforms play a role in development of the heart pre- and post-natally. Thus, mice lacking these isoforms show impaired myocardial angiogenesis (Carmeliet et al, 1999).

All of the variant VEGF proteins are assumed to be secreted in that they share the same secretion signal sequence of 26 amino acids. Compared with VEGF121, VEGF165 has an insertion of 44 amino acids rich in basic residues encoded by exon 7 and VEGF189 has a further insertion of 24 amino acids highly enriched in basic residues encoded by exon 6a. Despite this, little is known concerning the in vitro activities of the different isoforms. While the physiological significance of the differential splicing remains to be determined, we have examined the effects of the differential splicing on tumorigenesis. Several studies have reported transfection of VEGF into a range of carcinoma lines, followed by examination of the effect on tumorigenesis (reviewed in Bicknell et al, 1997). These studies uniformly concur that VEGF expression enhances tumour growth and some conclude that it also enhances the tumour vascular density. In the case of the

*Received 20 October 1999*
*Revised 15 February 2000*
*Accepted 19 February 2000*

*Correspondence to*: R Bicknell hormone-dependent MCF-7 cell line, VEGF121 expression has no effect on hormone dependence or tamoxifen sensitivity (Zhang et al, 1995). However, in none of these studies has the effect of expression of the 121, 165 and 189 isoforms on in vivo growth of capillary vessels and tumours been directly compared. This paper describes such a comparison.

### MATERIALS AND METHODS

### Vector construction

VEGF expression plasmids were constructed by inserting full-length complementary DNA (cDNA) into pcDNA1NEO (Invitrogen) in which the cytomegalovirus (CMV) promoter drives constitutive expression. The cDNAs of VEGF165 (a gift of H Weich, Braunschweig, Germany) and VEGF189 (a gift of J Abraham, Scios Nova, USA) were cloned as *Bam*H 1-*Eco*R V and *Bam*H 1 fragments respectively into the compatible sites of pcDNA1NEO. The resulting constructs were propagated in *Escherichia coli* strain MC1061/P3. The 5′ untranslated regions of the VEGF121 and 165 cDNAs were indentical and very similar to that of the VEGF189.

### Preparation of VEGF overexpressing MCF-7 cells

VEGF165 and VEGF189 overexpressing MCF-7 cells were prepared essentially as described for the preparation of VEGF121 overexpressing cells (Zhang et al, 1995). Clones were initially screened by ribonuclease protection analysis. The conditioned media from clones overexpressing VEGF message was then assayed for VEGF protein expression by enzyme-linked immunosorbent assay (ELISA). Serum-free medium was conditioned for 48 h by confluent cultures. Suramin (Zeneca Pharmaceuticals Alderly Edge, UK) was added to a concentration of 1 mM 4 h before harvesting to displace cell bound VEGF. ELISA was performed using a ‘Quantikine’ kit (R and D Systems, Abingdon, UK) according to manufacturer’s instructions.

#### *Effect of VEGF121 and VEGF165 on microvascular endothelial cell growth in vitro*

On day –1, human dermal microvascular endothelial cells (HDMECs) were seeded into collagen-coated 6-well plates at 2000 cells $cm^2$ in essential balanced medium (EBM) (Clonetics) containing epidermal growth factor (EGF) (10 ng $ml^{-1}$) and endothelial cell growth supplement (30 µg $ml^{-1}$). On day 0 the cells were counted and media in the remaining wells changed to EBM containing 5% fetal calf serum (FCS) and 8 ng $ml^{-1}$ VEGF121 or VEGF165 (both from R and D Systems, Minneapolis MN, USA). Cells were re-fed every other day or released by treatment with trypsin and counted in a Coulter counter (Coulter Electronics).

#### *Angiogenesis in vivo: rabbit cornea assay*

The cornea is an avascular and transparent tissue and growing capillaries may be easily monitored and quantitated by stereomicroscopic examination. Corneal assays were performed in female New Zealand albino rabbits (Charles River, Calco, Como, Italy) as described (Zhang et al, 1995) and in accordance to European Union animal care and welfare (EU law N. 86/609). MCF7 cells overexpressing the different VEGF isoforms were detached and resuspended at the density of $2.5 \times 10^5$ cells per 5 µl. The cell suspension in a volume of 5 µl was implanted into a corneal micropocket. Subsequent daily observation of the implants was made with a slit lamp stereomicroscope without anaesthesia. An angiogenic response was scored positive when budding of vessels from the limbal plexus occurred after 3–4 days and capillaries progressed to reach the implanted pellet according to the scheme previously reported. The number of positive implants over the total implants performed was scored during each observation. The potency of angiogenic activity was evaluated on the basis of the number and growth rate of newly formed capillaries, and an angiogenic score was calculated [vessel density × distance from limbus] as previously reported (Ziche et al, 1994, 1997).

### Xenograft experiments in BALBc *Nu/Nu* mice

A total of $10^7$ MCF-7 cells were co-xenografted with $10^7$ MDA-435S cells, exactly as described (Zhang et al, 1995). Xenograft experiments were performed in accordance with the British Home Office Animals (Scientific Procedures) Act of 1986. Tumour cytosols were prepared according to Smith et al (1993).

### Immunohistochemistry

Immunohistochemistry employed the VG1, anti-VEGF antibody that efficiently stains VEGF in paraffin sections (Turley et al, 1998). Immunohistochemistry was performed as described (Turley et al, 1998). Transfected cells were fixed, paraffin-embedded and stained exactly as for the tissues.

### Statistical analysis

*P*-values were calculated by the Mann–Whitney *U*-test (two-tailed).

### RESULTS

### Transfection of VEGF165 and VEGF189 into MCF-7 breast carcinoma cells

Plasmid containing the coding sequence for either VEGF165 or VEGF189 under the transcriptional control of the CMV promoter was transfected into MCF-7 breast carcinoma cells. VEGF-expressing clones were initially identified by overexpression of VEGF165 or VEGF189 mRNA (data not shown). Conditioned media from mRNA overexpressing clones was then screened for VEGF protein expression by ELISA. Before collecting media for ELISA, the cells were treated with media containing 1 mM suramin to release VEGF bound to heparin-like molecules on the cell surface into the media. Control assay of recombinant VEGF spiked with suramin showed that its presence did not interfere with the ELISA assay. Clones were chosen that, as far as possible, expressed similar amounts of the protein (Table 1), although the highest expressing 189 clone obtained expressed somewhat less protein than did transfectants of the other two isoforms.

### Immunohistochemistry of fixed, paraffin-embedded transfectants

Figure 1 shows immunostaining of control and transfected MCF-7 cells after fixation and paraffin embedding. Control and VEGF121 transfectants were negative. VEGF165 transfectants showed

 © 2000 Cancer Research Campaign

**Table 1** VEGF production by MCF-7 cells and transfectants in vitro determined by ELISA of conditioned media

| | CM – S | CM + S | Lysate |
|---|---|---|---|
| MCF-7wild-type | 3.1 | 6.0 | 0 |
| MCF-7NEO | 4.7 | 8.8 | 0 |
| MCF-7VEGF121 | 97.9 | 97.0 | 6.5 |
| MCF-7VEGF165 | 46.1 | 64.1 | 17.5 |
| MCF-7VEGF189 | 7.6 | 20.7 | 1.4 |

VEGF protein is given in ng ml$^{-1}$. Assays were carried out in quadruplicate. The +S conditioning/media contained 1 mM suramin to release VEGF165 and VEGF189 from the cell surface. CM, conditioned media. S, suramin.

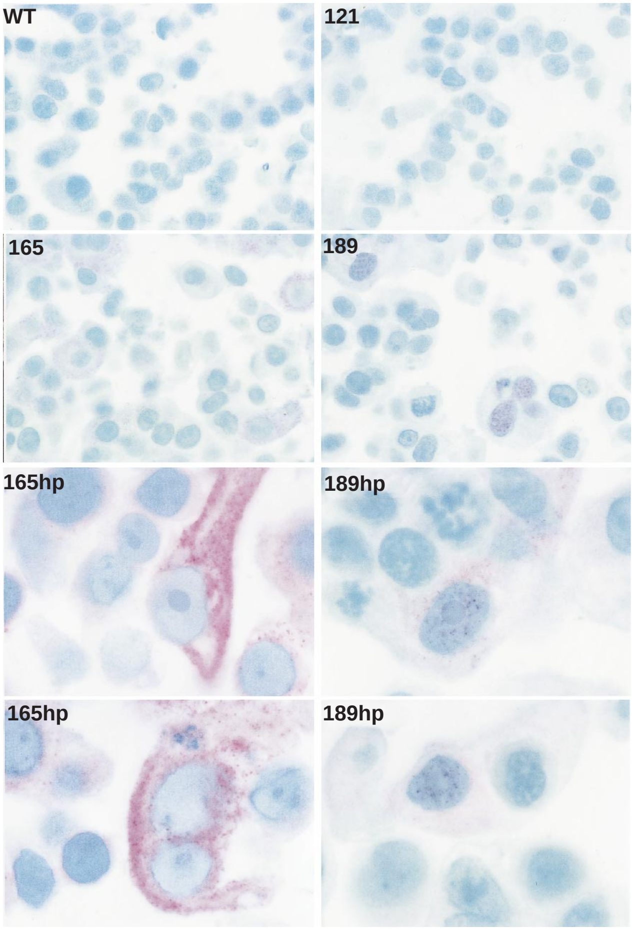

**Figure 1** Immunohistochemical staining of fixed, paraffin-embedded MCF-7 cell transfectants. All transfectants were treated identically (in parallel) in the same experiment to rule out artificial immunostaining of e.g. the nucleus strong cytoplasmic staining and the VEGF189 transfectants demonstrated nuclear staining. We have shown by Western analysis that the VG1 antibody recognizes VEGF121, VEGF165 and VEGF189 (Turley et al, 1998). It follows from this that it should recognize all three isoforms in paraffin sections equally.

### Effect of VEGF121 and VEGF165 on the growth of microvascular endothelial cells in vitro

Human dermal microvascular endothelial cells were stimulated with recombinant VEGF121 and VEGF165. Figure 2 shows that the in vitro growth stimulatory activity of these two isoforms was similar.

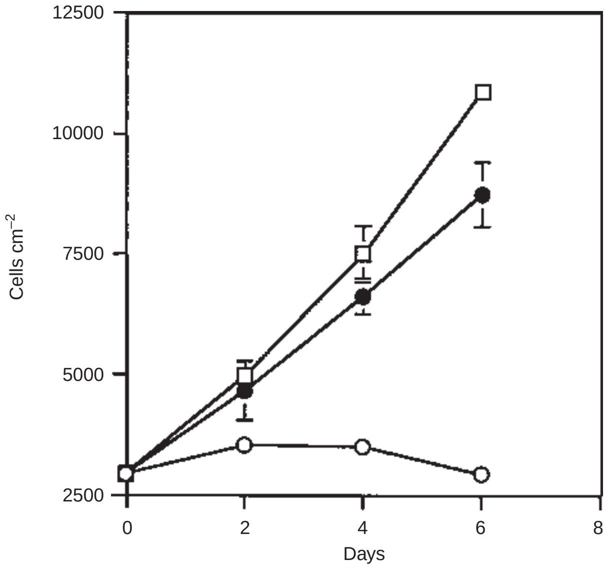

**Figure 2** Effect of recombinant VEGF121 and VEGF165 on the growth of human dermal microvacular endothelial cells in vitro (*n* = 2, ± s.d.). Control (○), VEGF121 (●) and VEGF165 (□)

### Effect of VEGF expression on MCF-7 cell growth in vitro

To compare the growth rates of the clones in vitro, cells were seeded at a density of 25 000 per well in quadruplicate in a 24-well plate. Cells were counted 1 week later as follows: MCF-7NEO 33 800 ± 1255, MCF-7VEGF121 33 500 ± 1480, MCF-7VEGF165 34 025 ± 895 and MCF-7VEGF189 33 200 ± 1055. We note that the growth rate of the cells appears unusually slow. This is not due to an intrinsically slow rate of growth but because these early passage MCF-7 cells undergo sustained apoptosis when cultured in vitro. The apoptosis is a feature of the early but not late passage cells. It is, however, clear that there was no significant difference in the increase in viable cell numbers in vitro between any of the four clones ($P > 0.05$).

### Rabbit corneal angiogenesis assays

Figure 3 shows that the MCF-7VEGF121 cells elicit a more rapid and extensive angiogenic response in the rabbit corneal angiogenesis assay than do control MCF-7NEO cells. In contrast, MCF-7VEGF165 and MCF-7VEGF189 showed a much weaker induction of angiogenesis in the assay. The response was more similar to that shown by control MCF-7NEO cells. More striking was the time taken to elicit a response following implantation. Thus, only the MCF-7VEGF121 cells elicited a rapid response, with detectable angiogenesis occurring on days 5–7 post implantation. In contrast, the other clones showed no response until at least 20 days post implantation. Nevertheless, between days 22 and 27 the response to MCF-7VEGF165 and MCF-7VEGF189 was greater than that in the control MCF-7NEO cells.

*© 2000 Cancer Research Campaign*

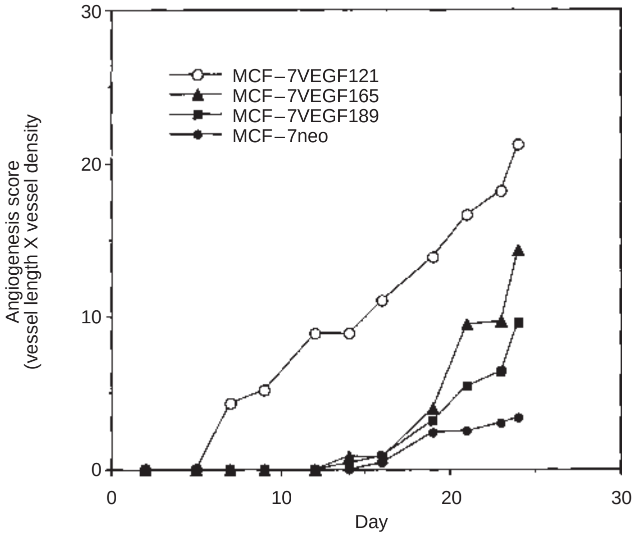

**Figure 3** Angiogenic activity of control MCF-7 cells and the VEGF expressing MCF-7 cell transfectants in the rabbit corneal assay.

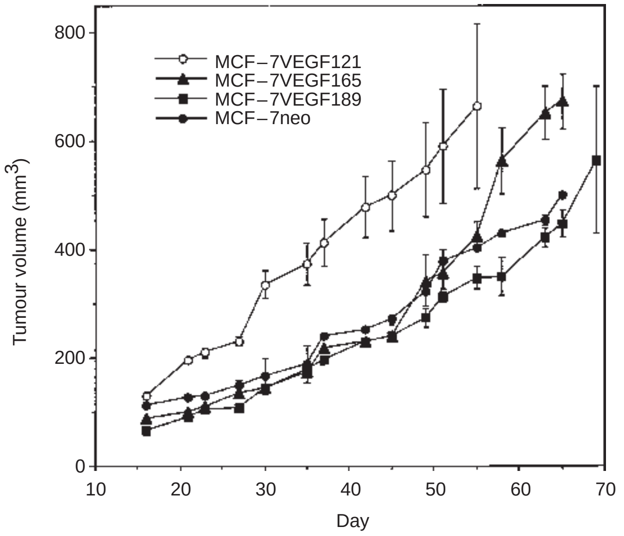

**Figure 4** Growth of MCF-7 xenografts in BALBc *nu/nu* athymic mice. $n = 10$ for each group ± s.d. The tumour volume in mm$^3$ is given (length × width$^2$)/2. A similar result was seen for 4 out of 5 experiments

### Xenograft of MCF-7 transfectants with MDA-435S breast carcinoma cells into BALBc *Nu/Nu* athymic mice

We have previously shown that early passage (< 65) MCF-7 cells are weakly tumorigenic when xenografted into nude mice, showing poor tumour take. To circumvent this problem we routinely co-xenograft our MCF-7 transfectants with the tumorigenic MDA-435S cells. Co-xenografting gives rise to a mixed tumour with islands of oestrogen receptor (ER)-positive MCF-7 cells growing within the ER-negative MDA-435S cells. This mimics the situation frequently found in primary human breast carcinomas where there is often a mixed population of ER-positive and ER-negative cells within the tumour. We have found this model to consistently give highly reproducible tumour growth. Thus, the four transfectants were co-xenografted with an equal number of MDA-435S cells into nude mice. Figure 4 shows the result of a typical experiment. It is seen that the MCF-7VEGF121 cells form more rapidly growing tumours than do wild-type cells. In contrast, VEGF165 or VEGF189 expression had a far less marked effect on tumorigenesis. In fact, VEGF189 expression showed no effect on tumour growth compared to controls, whereas VEGF165 did enhance tumor growth but only 50 days post xenograft. Table 2 gives the VEGF content of tumours determined by ELISA of tumour cytosols.

**Table 2** VEGF content of xenografted tumours determined by ELISA of homogenates

| | VEGF (pg mg$^{-1}$ protein) |
|---|---|
| MCF-7 neo | 255 ± 42.5 (*n* = 7) |
| MCF-7VEGF121 | 308.5 ± 42.5 (*n* = 5) |
| MCF-7VEGF165 | 13 501 ± 106.4 (*n* = 9) |
| MCF-7VEGF189 | 255 ± 53.2 (*n* = 8) |

*n* = number of tumours sampled. All tumours sampled were of very similar size.

### DISCUSSION

This study has aimed to compare the effects of the most commonly expressed isoforms of VEGF on tumorigenesis in a mouse model. We have used early passage MCF-7 cells because these are known to produce low levels of only a few angiogenic factors, including VEGF (Zhang et al, 1995). VEGF165 and VEGF189 were independently transfected into human MCF-7 breast carcinoma cells and clones selected that, as far as possible, expressed comparable amounts of the VEGF protein by ELISA. Selected clones were then examined in the rabbit corneal angiogenesis assay and in a mouse xenograft model.

Prior to the in vivo studies, the transfectants were fixed, paraffin-embedded and immunostained for VEGF. The 121 transfectant did not immunostain; however, there was much VEGF in the conditioned media from the ELISA analysis, even in the absence of suramin. We conclude that the 121 isoform is freely released from the MCF-7 cells. In contrast, immunostaining of the 165 transfectant showed there to be a substantial amount of VEGF165 retained in the MCF-7 cell cytoplasm. This was supported by an ELISA of the cell lysate (Table 1) that showed 17.5 ng ml$^{-1}$ of 165 versus 6.5 ng ml$^{-1}$ 121 and 1.4 ng ml$^{-1}$ of 189 respectively. Nevertheless, ELISA of the 165 isoform in suramin-containing conditioned medium confirmed that substantial amounts of VEGF165 were secreted. Retention of VEGF165 in the Golgi apparatus has been reported in other transfectants (Park et al, 1993). Although nuclear staining was seen with the 189 transfectant, again substantial VEGF was released in vitro by the cells (20.7 ng mg$^{-1}$ in the conditioned medium of suramin-treated cells, Table 1). Conditioned media from each of the transfectants was shown to have greater growth stimulatory activity on human dermal microvascular endothelial cells than did that from control empty vector transfectants (data not shown). Thus, we conclude that in each case the VEGF made by the transfectant is active. Figure 2 shows that VEGF121 and VEGF165 have similar growth stimulatory activity in vitro.

The rabbit corneal assay showed that the secretion of the VEGF121 isoform gave rise to much greater angiogenic activity than did secretion of the 165 or 189 isoforms. Consistent with this

*© 2000 Cancer Research Campaign*

**Table 3** Human VEGF189 contains a conserved nuclear localization motif found at the site of a number of known nuclear targeting sequences

| Protein | | Sequence |
|---|---|---|
| Nucleoplasmin (xenopus) | 155 | **KR**paatKKagqa**KKKK**1 |
| HIF-1alpha (human) | 18 | **RR**KeKsRdaaRs**RR**s**K**e |
| HIF-1alpha (mouse) | 18 | **RR**KeKsRdaaRs**RR**t**K**e |
| VEGF189 (human) | 115 | **KK**svRgKgKgq**KRKRKK**sRyKswsv |
| VEGF188 (mouse/rat) | 114 | **KK**svRgKgKgq**KRKRKK**sRfKswsv |
| VEGF – (chicken/quail) | – | **KK**sKRgKgKgq**KRKRKK**gRyKppsf |
| HIF-1alpha (human) | 719 | **KRKRK** |
| HIF-1alpha (mouse) | 715 | **KRKRK** |
| SV40 T antigen(virus) | – | **KKKRK** |

The amino acid sequence deduced from exon 6 of the human VEGF gene and its counterparts in other species are aligned with bipartite basic domains in *Xenopus* nucleoplasmin nuclear targeting sequence (Robbins et al, 1991). A similar sequence motif occurs frequently in nuclear proteins but rarely in cytoplasmic proteins. In addition, a number of the currently known targeting sequences have a strikingly similar organization (Robbins et al, 1991).

it was found that the 121 isoform enhanced tumorigenesis much more so than did expression of the 165 isoform, although the latter did enhance tumour growth 50 days post xenograft. Expression of the VEGF189 isoform had no effect on tumour growth. It is known that the different isoforms bind to the VEGF receptors flt-1 and KDR with equal affinity and that they have the same growth stimulating activity but that they differ in their affinity for heparin or heparin-like molecules. It is postulated that it is this different affinity for heparin like molecules that leads to the different effects on tumourigenenesis. The in vitro data show that VEGF165 and VEGF189 but not VEGF121 are tightly bound to the extracellular surface of the MCF-7. Thus, VEGF165 and VEGF189 are released from the cell surface in significant amounts only on treatment with a heparin competitor such as suramin (Table 1). If the same were to happen in vitro it would explain the observations. Thus, in the rabbit cornea, VEGF121 freely diffuses through the cornea away from the MCF-7 cell implant toward the vascular limbus, where it initiates angiogenesis. Compare this to the 165 and 189 isoforms which remain bound to the surface of the MCF-7 cells and take 20 days post implant to reach the vascular limbus. It is notable that when the angiogenic response to VEGF165 or VEGF189 does finally occur it is as strong and rapid as that to VEGF121, consistent with the isoforms being equipotent in endothelial growth factor activity (Figure 2). The response to the 165 and 189 transfectants was similar despite the greater release of VEGF165 than VEGF189.

Several growth factors, thought to be extracellularly acting, have subsequently been shown to localise to the nucleus (e.g. basic fibroblast growth factor (bFGF) (Coltrini et al, 1995) the related int-2 (Acland et al, 1990) and interferon-γ (Bader and Wiezerbin, 1994). The biological function of this localization has remained unknown, particularly in the light of the fact that the bFGF transfectants show no known phenotypic differences (Coltrini et al, 1995). In the case of bFGF and int-2 the subcellular fate of the protein was shown to depend on the choice of initiation codon. VEGF is different in that the subcellular localisation is determined not by choice of initiation codon but by differential splicing. A strong consensus nuclear localisation signal was identified in exon 6a of VEGF (Table 3) (Robbins et al, 1991) and only isoforms containing this exon (145, 189 and 206) will have the nuclear localization signal. There are no nuclear localization signals in any of the other growth factors showing sequence homology to VEGF (i.e. VEGF-B, C, D, E or PLGF, PDGF-A and PDGF-B) (data not shown).

The overall conclusion of this study is that the 121 shows a stronger induction of tumorigenesis than do the other commonly expressed 165 and 189 isoforms in vivo. This is particularly significant in view of the fact that the 121 isoform has been shown to predominate in primary human breast carcinomas (Relf et al, 1997). It also has important implications for clinical applications in which the stimulation of angiogenesis is the desired objective, e.g. ischaemic limb re-vascularization. As VEGF121 is freely diffusible, its use in such studies has clear advantages. The reason for selective nuclear localisation of VEGF189 is, as for other factors that show a similar effect, completely unclear.

### ACKNOWLEDGEMENTS

This work was supported by the Imperial Cancer Research Fund and funds from the Italian Association for Cancer Research (AIRC), European Communities BIOMED-2: 'Angiogenesis and Cancer', and Italian Ministry of University, Scientific and Technological Research (MURST) to MZ.

Prudence Scott was in receipt of funding from the Health Research Council of New Zealand and Imperial Cancer Research Fund, and later the Nuffield Trust.

### REFERENCES

Acland P, Dixon M, Peters G and Dickson C (1990) Subcellular fate of the int-2 oncoprotein is determined by choice of initiation codon. *Nature* **343**: 662–665

Bader T and Wiezerbin J (1994) Nuclear accumulation of interferon-γ. *Proc Natl Acad Sci USA* **91**: 11831–11835

Bicknell R (1997) Mechanistic insights into tumour angiogenesis. In: *Tumour Angiogenesis*, Bicknell R, Lewis CE and Ferrara N (eds), pp. 19–28. Oxford University Press: Oxford

Carmeliet P, Ng Y-S, Nuyens D, Theilmeier G, Brusselmans K, Cornelissen I, Ehler E, Kakkar VV, Stalmans I, Mattot V, Perriard J-C, Dewerchin M, Flameng W, Nagy A, Lupu F, Moons L, Collen D, D'Amore PA and Shima DT (1999) Impaired myocardial angiogenesis and ischemic cardiomyopathy in mice lacking the vascular endothelial growth factor isoforms VEGF164 and VEGF188. *Nature Med* **5**: 495–502

Cheung N, Wong MP, Yuen ST, Leung SY and Chung LP (1998) Tissue-specific expression pattern of vascular endothelial growth factor isoforms in the malignant transformation of lung and colon. *Hum Pathol* **29**: 911–914

Coltrini D, Gualandris A and Nelli EE (1995) Growth advantage and vascularisation induced by basic fibrolblast growth factor overexpression in endometrial HEC-1-B cells: An export -dependent mechanism of action. *Cancer Res* **55**: 4729–4738

Gimbrone MA Jr, Cotran RS, Leapman SB and Folkman J (1974) Tumor growth and neovascularisation: an experimental model using the rabbit cornea. *J Natl Cancer Inst* **52**: 413–427

Gitay-Goren H, Cohen T, Tessler S, Soker S, Gengrinovitch S, Rockwell P, Klagsbrun M, Levi BZ and Neufeld G (1996) Selective binding of VEGF121 to one of the three vascular endothelial growth factor receptors of vascular endothelial cells. *J Biol Chem* **271**: 5519–5523

Grutzkau A, Kruger-Krasagakes S, Baumeister H, Schwarz C, Kogel H, Welker P, Lippert U, Henz BM and Moller A (1998) Synthesis, storage and release of vascular endothelial growth factor/vascular permeability factor (VEGF/VPF) by human mast cells: implications for the biological significance of VEGF206. *Mol Biol Cell* **9**: 875–884

Houck KA, Ferrara N, Winer J, Cachianes G, Li B and Leung DW (1991) The vascular endothelial growth factor family: identification of a fourth molecular species and characterisation of alternative splicing of RNA. *Mol Endocrinol* **5**: 1806–1814

Houck KA, Leung DW, Rowland AM, Winer J and Ferrara N (1992) Dual regulation of vascular endothelial growth factor bioavailability genetic and proteolytic mechanisms. *J Biol Chem* **267**: 26031–26037

© 2000 Cancer Research Campaign

Keyt BA, Berleau LT, Nguyen HV, Chen H, Heinsohn H, Vandlen R and Ferrara N (1996) The carboxyl-terminal domain (111–165) of vascular endothelial growth factor is critical for its mitogenic potency. *J Biol Chem* **271**: 7788–7795

Lei J, Jiang A and Pei D (1998) Identification and characterisation of a new splicing variant of vascular endothelial growth factor: VEGF183. *Biochim Biophys Acta* **1443**: 400–406

Neufeld G, Cohen T, Gengrinovitch S and Poltorak Z (1999) Vascular endothelial growth factor and its receptors. *FASEB J* **13**: 9–22

Park JE, Keller GA and Ferrara N (1993) The vascular endothelial growth factor (VEGF) isoforms: differential deposition into the subepithelial extracellular matrix and bioactivity of extracellular matrix-bound VEGF. *Mol Biol Cell* **4**: 1317–1326

Relf M, LeJeune S, Scott PAE, Fox S, Smith K, Leek R, Moghaddam A, Whitehouse R, Bicknell R and Harris AL (1997) Expression of the angiogenic factors vascular endothelial cell growth factor, acidic and basic fibroblast growth factor, transforming growth factor β-1, platelet-derived endothelial cell growth factor, placenta growth factor and pleiotrophin in human primary breast cancer and its relation to angiogenesis. *Cancer Res* **57**: 963–969

Robbins J, Dilworth SM, Laskey RA and Dingwall C (1991) Two independent basic domains in nucleoplasmin nuclear targeting sequence: Identification of a class of bipartite nuclear targeting sequence. *Cell* **64**: 615–623

Siemeister G, Schnurr B, Mohrs K, Schachtele C, Marme D and Martiny-Baron G (1996) Expression of biologically active isoforms of the tumor angiogenesis factor VEGF in Escherichia coli. *Biochem Biophys Res Commun* **222**: 249–255

Smith K, Houlbrook S, Greenall M, Carmichael J and Harris AL (1993) Topoisomerase IIα coamplification with erbB-2 in human primary breast cancer and breast cancer cell lines: relationship to m-AMSA and mitoxantrone sensitivity. *Oncogene* **8**: 933–938

Tischer E, Mitchell R, Hartman T, Silva M, Gospodarwicz D, Fiddes JC and Abraham JA (1991) The human gene for vascular endothelial growth factor. Multiple protein forms are encoded through alternative exon splicing. *J Biol Chem* **266**: 11947–11954

Turley H, Scott PAE, Watts VM, Bicknell B, Harris AL and Gatter KG (1998) Expression of VEGF in routinely fixed material using a new monoclonal antibody VG1. *J Pathol* **186**: 313–318

Zhang H, Craft P, Scott PAE, Ziche M, Weich HA, Harris AL and Bicknell R (1995) Enhancement of tumor growth and vascular density by transfection of vascular endothelial cell growth factor into MCF-7 human breast carcinoma cells. *J Natl Cancer Inst* **87**: 213–219

Ziche M, Morbidelli L, Masini E, Amerini S, Granger HJ, Maggi CA, Geppetti P and Ledda F (1994) Nitric oxide mediates angiogenesis in vivo and endothelial cell growth and migration in vitro promoted by substance P. *J Clin Invest* **94**: 2036–2044

Ziche M, Morbidelli L, Choudhuri R, Zhang H-T, Donnini S, Granger HJ and Bicknell R (1997) Nitric oxide-synthase lies downstream of vascular endothelial growth factor but not basic fibroblast growth factor induced angiogenesis. *J Clin Invest* **99**: 2625–2634

*© 2000 Cancer Research Campaign*
